# Role of Anillin in Tumour: From a Prognostic Biomarker to a Novel Target

**DOI:** 10.3390/cancers12061600

**Published:** 2020-06-17

**Authors:** Nguyen Minh Tuan, Chang Hoon Lee

**Affiliations:** College of Pharmacy, Dongguk University, Seoul 04620, Korea; tuank67a5@gmail.com

**Keywords:** anillin, cytokinesis, actin-binding protein, tumour microenvironments, inhibitors

## Abstract

Anillin (ANLN), an actin-binding protein, reportedly plays a vital role in cell proliferation and migration, particularly in cytokinesis. Although there have been findings pointing to a contribution of ANLN to the development of cancer, the association of ANLN to cancer remains not fully understood. Here, we gather evidence to determine the applicability of ANLN as a prognostic tool for some types of cancer, and the impact that ANLN has on the hallmarks of cancer. We searched academic repositories including PubMed and Google Scholar to find and review studies related to cancer and ANLN. The conclusion is that ANLN could be a potent target for cancer treatment, but the roles ANLN, other than in cytokinesis and its influence on tumour microenvironment remodeling in cancer development, must be further elucidated, and specific ANLN inhibitors should be found.

## 1. Introduction

Anillin (ANLN) is an actin-binding protein that has been documented as a key factor in cell division, and it is a multi-domain protein that interacts with many proteins [1]. ANLN is highly expressed in many types of site-specific cancerous tumours, including brain, lung, pancreas, and bone marrow cancer [2]. To date, many studies have emphasized the involvement of ANLN in cancer progression, including pancreatic, colorectal, breast, and lung cancers [3,4,5,6,7,8,9]. While the tumour microenvironment is considered to be a key contributor to the development of cancer, there is scant research as to the impact of ANLN on the characteristics of the microenvironment, particularly on inflammation and immunity. In addition, the contributors to the particular characteristics of the cancer cells, such as proliferation and migration, have drawn more attention. Despite ANLN being present in different locations ranging from the nucleus to the cortex and having interactions with many proteins [10,11], its role has mostly been described to be related to cytokinesis with its presence in the midbody. However, some studies have shown an association between cancer development and the level of ANLN expression in the nucleus, as well as the role of ANLN outside a cytokinesis event.

Hence, the review starts by describing the physiological role, summarizing its main interaction partners, its role in cancer, and ends with biomarkers and potential therapeutic options. We hope that based on this review, a research direction can be further clarified for ANLN.

## 2. Role of ANLN in Normal Cell

### 2.1. Findings from Drosophila and C. elegans

ANLN was isolated as an F-actin-binding and bundling protein from Drosophila melanogaster embryo extracts [12]. ANLN localizes to the nucleus during interphase, to the cortex upon nuclear envelope breakdown, to the cleavage furrow in anaphase and to the midbody rings during telophase and into the next cell cycle [12].

RNAi (RNA interference)-mediated depletion of ANLN causes furrow instability in D. melanogaster S2 cells [13]. In these cells, furrows form normally at the cell equator, but then oscillate back and forth across the equator, parallel to the spindle axis. The loss of ANLN also promotes membrane blebbing and, in cases where a relatively stable furrow forms, a loss of stability of the midbody structure that forms after furrowing in D. melanogaster [14]. In the first division of Caenorhabditis elegans embryos, ANLN depletion does not appear to prevent cytokinesis but causes a loss of furrow asymmetry [15].

**Figure 1:**
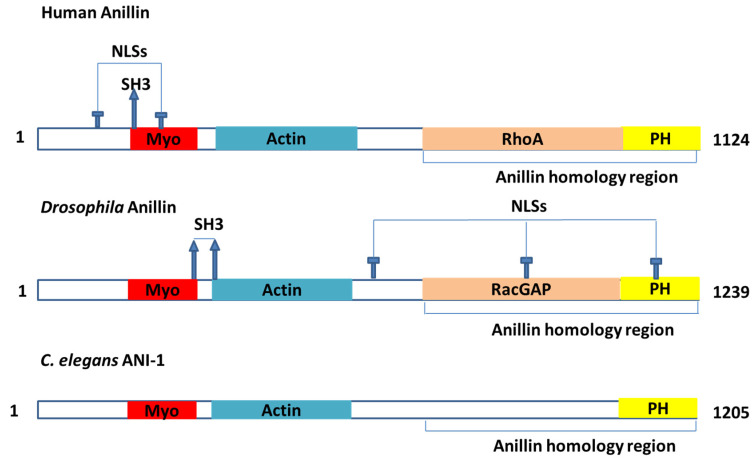
The model of the major domains in the ANLN actin-binding protein. My, myosin-II-binding domain; Actin, actin-binding domain; NLS, nuclear localization signal; PH, pleckstrin homology domain; RacGAP, region that interacts with the RacGAP50C component of the centralspindlin complex; RhoA, region that interacts with the GTPase RhoA; SH3, Src-homology-3-binding consensus sequences.

ANLN is a highly conserved protein that contains multiple domains [16] (Figure 1). These domains were documented to interact with many partners (Table 1), such as actin, myosin II and septins [1]. ANLN could act as an indispensable factor to scaffold and organize the cytoskeleton and its partners in the events related to cytokinesis [11,12,17]. ANLN is majorly associated with cortical cytoskeletal dynamics during cytokinesis and cellularization. However, other roles in different contexts are possible to be masked by their fundamental functions [11].

### 2.2. Binding Partners

ANLN, a conserved multi-domain protein interacting with many biological partners, is a prime factor that may act as a scaffold and be involved in organizing the cytoskeleton, as well as be a regulator in the entire aforementioned events.

#### 2.2.1. Binding Partners Related to Cytokinesis

ANLN interacts with some proteins during cytokinesis (Table 1). Among its partners, there are some proteins proven to be associated with cancer. Take actin, myosin, septins, RhoA, and RacGAP for example. 

**Actin**: Actin is a protein that is abundant in mammalian cells, and it is associated with the motility and compartmentalization of cellular contents. In eukaryotic cells, there are two main actin forms: globular G-actin and fibrillar F-actin. F-actin is composed of G-actin. G-actin can polymerize in the absence of associated proteins after ATP hydrolysis and Mg^2+^ consumption in vitro. However, in cells, actin polymerization is accelerated by actin nucleation factors such as Arp2/3 and formins [36]. The growth of these actin filaments is regulated by thymosin and profilin. Thymosin binds to G-actin to buffer the polymerizing process, while profilin binds to G-actin to exchange ADP for ATP, promoting the monomeric addition to the barbed, plus end of F-actin filaments [36,37]. Filaments are assembled and structured by actin-filament-bundling proteins [38]. ANLN was characterized as a molecule that is specifically associated with F-actin [18]. ANLN interacts with three types of actin filaments [12,23,39]. ANLN bundles actin filaments with two domains, including the amino acids 258–340 of Drosophila. ANLN was also described as a binding site for F-actin and amino acids 246–371 bundle actin filaments. The F-actin-binding domain of ANLN was recorded with two F-actin-binding sites, but one of them could shrink after forming a binding with F-actin [12]. F-actin and ANLN are independently attracted to the contractile ring in human and *Drosophila* cells [13,19,20,21]. Nevertheless, F-actin has a role in supporting ANLN to locate in the cell equator with accurate timing and spatial positioning [13,21,22]. More compellingly, ANLN protein binds specifically to the contractile ring in the cell division stage [1]. Studies with Drosophila spermatocytes have shown that the depletion of ANLN could lead to a deviation of F-actin, myosin-like protein out of the equator that induces a failure of cytokinesis [1,19]. In addition to the direct interaction with F-actin, ANLN also establishes an indirect interaction with F-actin through formin mDia2 binding, which could play a role in the stabilization of formin in the active configuration after binding RhoA [40]. The interaction between formin and ANLN has an indispensable role in the cortical localization of mDia2, contributes to successful cytokinesis [25] and seems to unravel the similar oscillation phenomenon taking place in the two cases of ANLN loss and mDia2 depletion [25]. This indicates that ANLN could be implicated in both actin organization and polymerization in the contractile ring [1].

As mentioned above, the ANLN locations vary from the nucleoplasm to the cytoplasm during a cell cycle, and ANLN is a multiple-domain scaffold. Taken together, this suggests that ANLN could have a direct or indirect impact on actin activities both outside and inside cytokinesis events, which could be related to cancer progression.

**Myosin:** ANLN could form an interaction with myosin, directly or indirectly [1]. ANLN may indirectly impact myosin through F-actin [1]. There has been evidence showing that in Drosophila and *C. elegans*, ANLN (ANI-1 in *C. elegans*) and myosin are attracted to the contractile ring independently [13,25,31]. The main function of ANLN to myosin is to organize them. This was observed in a finding of Haglund K in 2010, which found that ANLN in Drosophila is necessary and sufficient to organize myosin into rings during cellularization [41]. During cytokinesis, there is a disruption of the stability of timing and space at the cell equator of ANLN-depleted cells (human and Drosophila) [13,16,19,21,31,42]. *C. elegans* ANLN was documented to be involved in the organization of myosin into dynamic foci within the span of the polarity formation and cytokinesis [25,43] and encourages asymmetric furrow ingression located at the zygote [27]. *C. elegans* ANLN (ANI-2) is required for the integrity of the myosin [25]. Notably, the upregulation of ANLN could improve the efficacy of non-muscle myosin II by coordination between myosin and bundled F-actin, as well as increasing the likelihood in binding to the actin track [1]. Lateral oscillation during the polar regions of the cell could contribute to cytokinesis failure by abnormal contractile behaviour and can make the cytoplasm deviate from the equator [16]. The abnormality of the contractile behaviour in the polar region seems to be rooted in the mislocalization of the myosins located in the outside of the cell equator during oscillations. 

**Septins:** Septins belong to a group of GTP-binding proteins [44,45]. Different septins make a complex with one another [44]. These complexes can assemble into filaments and rings functioning as a fourth cytoskeleton [46]. ANLN was documented as a partner that binds to septins [1,46,47], a conserved family of GTP-binding proteins [48]. Septins are also recruited by ANLN to the contractile ring [24]. There was a direct interaction between the septins and ANLN identified in vitro [23]. The third C-terminal was characterized as a binding part to septins, whereas the third C-terminal was constituted by the terminal PH domain and the ANLN homology (AH) [1]. ANLN truncation occurs in human cells without the AH domain, which was considered as a factor mediating the interaction of septins, and showed the mislocalization of the poles in the span of oscillation like the event witnessed as a lack of myosin [16]. There was another ANLN truncation model without the PH domain, which was thought to be associated with septins and with a loss in the ability to localize to the cortex. The localization was still defective, even with endogenous ANLN presence [16]. In contrast to this, *C. elegans* ANLN recruitment in the contractile ring was independent of septin enrichment [25], but septins were necessary to asymmetrically localize ANLNs correctly for ring shrinkage [15]—all of which suggests an association between septins and ANLNs that bolsters the connection between the actin cytoskeleton and membrane [1]. 

**RhoA**: RhoA is a protein involved in multiple cellular processes that plays a central role in the regulation of actin organization, cell migration, cytokinesis, cell cycle regulation and cell proliferation [49,50,51,52]. The human AH domain could act as a bridge between ANLN and RhoA [53,54]. ANLN and RhoA were discovered to be co-immunoprecipitated [29]. Furthermore, the upregulation of ANLN could induce a significant increase in the rate of active RhoA [29]. The equatorial cortical localization of RhoA tended to be regulated by ANLN during cytokinesis because ANLN is associated with the cell membrane in both ways of direction or indirection via septins [1]. ANLN and RhoA were documented to have locations in proximity with each other [1]. The AH domain in the C-terminus of ANLN is able to bind directly to RhoA in vitro, and the role of this domain was proven to stabilize RhoA localization in vivo [16]—all of which could indicate that ANLN could be associated with activating RhoA and stabilizing it in the cleavage plane [1]. The interaction between ANLN and Ect2 (an activator of RhoA) was found, which supports the postulation about ANLN involved in regulating and stabilizing RhoA location [1].

**RacGAP**: ANLN was recorded to interact directly with RacGAP50C [1,30], which plays a role in specifying the cleavage site. This interaction was confirmed by a yeast two-hybrid assay. In another study, RacGAP was discovered to interact with full-length ANLN via amino acids 83–309 while the sequence of amino acids from 245 to 311 of RacGAP plays a role in the interaction with ANLN [30]. The half of the ANLN N-terminal of ANLN was observed not to interact with any RacGAP constructs. Specific RacGAP deletions to abolish Pebble or MKLP1 binding was documented without any effect on the interaction with ANLN [55]. The absence of ANLN leads to a loss of connection between the spindle-associated RacGAP and the equatorial cortex and to cytokinesis failure [30]. ANLN was also documented to interact with [56] and co-express [57] RacGAP1. 

#### 2.2.2. Other Binding Partners 

**Kinases**: ANLN was proved to be phosphorylated in early mitosis [33]. Forty-six phosphorylation sites of ANLN were discovered, but only phosphorylation at S635 was proven as an important requirement for the success of ANLN recruitment to cleavage furrow. This was also underpinned by the evidence of phosphomimetic-mutant S635D, the negative charge of D at the 635 residues which partially recovered the localization. S635 phosphorylation helps ANLN improve the efficacy of the Rho integration with its upstream and downstream regulators, which contributes to the success of cytokinesis [58]. To date, the kinases that are responsible for S635 remain unidentified, so there is a need to determine which kinases are responsible for the phosphorylation at S635. 

CDK1 was found to interact with ANLN [56,57], and it is indicated that ANLN mobility is directly or indirectly regulated by CDK1 via phosphorylation [58]. The ANLN-actin-binding protein has been identified as being involved in PI3K/PTEN signaling, which is critical in cell life/death control [59]. CITK was proven to be associated with the localization of F-actin and ANLN at the abscission sites [60].

**KIAA1429**: KIAA1429, a complex that is involved in the regulation of the N6-methyladenosine (m6A) methylation of RNAs, is a modification that plays a role in the effectiveness of mRNA splicing and RNA processing [61]. The interaction between KIAA1429 and ANLN was found by methods including affinity capture-mass spectrometry (MS) and affinity capture-RNA [62], and it is curated by BIOGRID [63]. KIAA1429 has recently been recognized as an oncogenic factor in several cancer types, including breast cancer, by regulating CDK1 [64]. KIAA1429 was also found to be associated with the migration and invasion of hepatocellular carcinoma by altering the m6A modification of ID2 mRNA [65]. The contribution of KIAA1429 to liver cancer progression was recorded [66]. The interaction between KIAA1429 and ANLN could suggest that we could monitor KIAA1429 expression through ANLN.

**MYC**: Myc is a well known family of regulator genes and proto-oncogenes that code for transcription factors. The Myc family includes three related human genes: c-Myc, l-Myc, and n-Myc. With a possible role in cancer, c-Myc is often expressed. The protein encoded by the Myc gene could act as a multifunctional protein that could be associated with cell growth, cellular transformation, and apoptosis [67]. The upregulation of Myc could be present in various types of cancers, such as colon, breast, lung and gastric cancer [68]. To date, Myc and ANLN have been discovered to interact through experimental evidence, including affinity capture-MS [69], and proximity label-MS [70] and curated by the Biogrid [63].

**KDR**: Kinase insert domain receptor (KDR) is a primary vascular endothelial growth factor receptor that encodes a crucial receptor regulating the cancer angiogenesis/metastasis switch [59]. KDR is also a key factor in controlling the survival, growth and migration of endothelial cells. Its upregulation has been found in various types of cancer cells [71]. ANLN has been identified as an interactor with KDR that encodes a key receptor mediating the cancer angiogenesis/metastasis switch. The observations have suggested that ANLN acts as the intrinsic connection between PI3K/PTEN and KDR signaling, which represents two critical transitions in carcinogenesis [59]. The interaction between ANLN and KDR could act as ANLN and KDR jointly as a prognostic in cancer survival, which could be applied to control triple negative breast cancer [71].

**CDC5L**: CDC5L is a DNA-binding protein that is implicated in cell cycle control and could act as a transcription factor. It takes a role in pre-mRNA splicing as a core factor of precatalytic, catalytic and post catalytic spliceosomal complexes [72,73,74,75,76,77,78,79]. The depletion of CDC5L leads to the inhibition of mitotic progression, and it induces mitotic failure [80]. Additionally, the downregulation of CDC5l inhibits the proliferation of bladder cancer cells [81]. ANLN has been experimentally proven to interact with CDC5L [82]. CDC5L is also located in the nucleus [83] where ANLN is primarily present during the cell cycle. In this regard, ANLN is likely to play a role in the splicing involving CDC5L.

**TAF10**: Gene ontology annotations associated with the TATA-box-binding protein associated factor 10 (TAF10) are the DNA-binding transcription factor activity and the transcription coactivator activity. TAF10 is involved in the process of Drosophila erythropoiesis via the GATA1 transcription factor [84], and it appears to play a dispensable role in the somitogenesis process and the Drosophila morphogenesis process in mice [85,86]. TAF10 inactivation in liver tissue was observed to dissociate TFIID complexes individually, but genes affected by TAF10 inactivation were less than 5% of the active genes [87]. ANLN interacts with TAF10 [88] and is present in the nucleus when it is in interphase cells and syncytial embryo [12]. It seems that ANLN is likely to function in relation to the role of TAF10.

**BRCA1**: BRCA1 is essentially involved in the repair of double-strand breaks by recruiting DNA repair enzymes. Loss of function mutations decrease the repair of DNA double-strand breaks and thereby increases the mutation frequency and the risk of cancer [89]. The encoded protein combines with other tumour suppressors, DNA damage sensors, and signal transducers to form a large multi-subunit protein complex known as the BRCA1-associated genome surveillance complex (BASC) [90]. The BRCA1 mutation is found in breast cancer in young women, which is a triple negative breast cancer [91]. ANLN interacts with BRCA1 [92]. Therefore, there is a possibility that ANLN modulates DNA repair by interacting with BRCA1 in the nucleus.

**Others including microtubules**: Drosophila ANLN has an affinity with microtubules [26]. The alignment of spindle-microtubules in metaphase was regulated by microtubule ANLN interaction [1]. In addition, human ANLN was documented to interact with astral microtubules [93]. Astral microtubules and spindle microtubules may independently restrict the localization of ANLN and other contractile ring proteins at the equatorial cortex. This sequestration could change the organization of cortical proteins to polarize cells in cytokinesis [93]. Some other potential interactors with ANLN were discovered and curated by String and Biogrid, such as CALML3, KIF 23, KIF20A [57,63]. 

### 2.3. Role of ANLN during Cytokinensis

**Figure 2:**
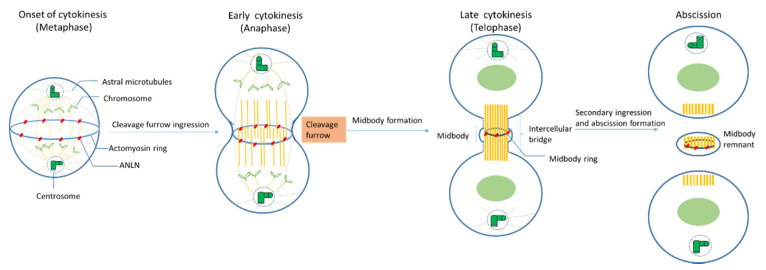
The roles of ANLN during cytokinesis.

During mitosis, ANLN locations shift drastically (Figure 2). In vivo immune-staining observations of cultured *Drosophila* cells and human cells showed that ANLN resides in nuclei during the interphase and relocates to contractile rings during cytokinesis [12,22,39,42].

**Onset of cytokinesis (metaphase):** When nuclear envelopes are broken down, ANLN moves from the nuclei to peripheral stress fibers in mammalian cells [23], at which ANLN might mediate these fibers’ disassembly and increase the round cortex of *Drosophila* cells [13,30]. In *S. pombe*, Mid1 (an ANLN-like protein) locates at the equatorial cortex before any other contractile ring component and acts as a scaffold for other components such as RhoA, actin, myosin, and the septins [1]. ANLN depletion leads to a mislocalization of F-actin, like myosin, out of the equator in *Drosophila* spermatocytes, which could lead to a failure in cytokinesis [19]. In *C. elegans*, ANLN (ANI-1) is associated with an asymmetry of the division plane, which plays an important role in the perturbations of contractility during cytokinesis [15]. Metazoan ANLN coordinates contractile ring assembly and organization by crosslinking with myosin septins and F-actin in the actin–myosin ring [1]. 

**Early cytokinesis (anaphase):** At the beginning of anaphase, the mitotic spindle forms a dense array of antiparallel microtubules called the central spindle [1]. The ingression of the plasma membrane at the cell equator occurs and a cleavage furrow is formed. Although actin–myosin contractility still happens in both human and *Drosophila* cultured cells that have been depleted of ANLN, the depletion of ANLN leads to the lateral oscillation of cleavage furrow, or its failure cannot accurately maintain at the division plane [13,19,27,31,42]. The knockdown of ANLN in human cells and *Drosophila* ANLN mutants caused slow and abortive furrowing as well as slow ingression [1]. However, there are some findings showing that ANLN function in furrow initiation and ingression could be compensated for other mechanisms such as MKLP1 (a component of contractile spindle), ZEN-4 (homogenous kinesin), and cadherins [1]. 

**Late cytokinesis (telophase):** At late cytokinesis, the actin and myosin contractile ring that separates daughter cells may assemble through interaction with at least two other furrow proteins, actin, and septins [22]. ANLN-depleted *Drosophila* cells unveiled that ANLN could function in the stabilization of the midbody because the depletion of ANLN in cells could lead to a reduction in microtubule integrity in the midzone and to blebbing around the midbody which is the central region of the thin intercellular cytoplasmic bridge formed between daughter cells during cytokinesis. It consists of tightly bundled antiparallel microtubules, which embraces a phase-dense circular structure, called the midbody ring [14,94]. There is evidence suggesting that the localization of ANLN at this late stage plays an important role in the completion of cytokinesis [95].

**Abscission:** Before abscission, ANLN and F-actin were maintained at secondary ingression by abscission regulator citron kinase (CITK), and the F-actin localization at secondary ingression sites is required for normal abscission [60], which indicates that ANLN may be relevant for this function. At the final stage of cytokinesis, the intercellular bridge is cleaved in a process termed abscission, and two daughter cells are formed. Following abscission, the residual midbody structure, known as the midbody remnant or midbody derivative, can have different fates depending on the cell type. It can either be released into the extracellular medium, be degraded by autophagy, or persist in the cytoplasm, showing asymmetric accumulation in the daughter cells [36].

### 2.4. Mechanism of ANLN-Controlled Cytoskeletal Dynamics

**ANLN binds to F-actin:** ANLN binds to F-actin only during the cell division process. ANLN forms a bundle of F-actin filaments [12]. By controlling actin bundling, ANLN increases the efficiency of actomyosin contractility during cell division. ANLN and F-actin are independently recruited as contractile rings, but F-actin increases the efficiency of ANLN summons [1]. ANLN also promotes the polymerization of F-actin through stabilizing formin mDia2 in active form. [40].

**Figure 3:**
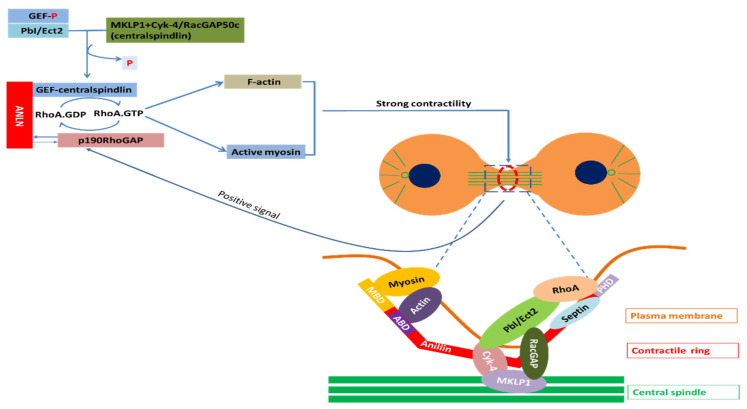
Partners of ANLN in cytokinesis; Myosin, a superfamily of motor proteins; Actin, a family of globular multi-functional proteins that form microfilaments; RhoA, a small GTPase protein in the Rho family of GTPases; Septin, a group of GTP-binding proteins; Cyk-4, a Rho family GTPase-activating protein (Gap) required for central spindle formation and cytokinesis; MKLP1, a member of the kinesin-like protein family; RhoA activation: Ect2, epithelial cell transforming 2 in mammalian cells or PbI in *Drosophila*; ANLN, anillin actin-binding protein; MBD, myosin-binding domain; ABD, actin-binding domain; PHD, pleckstrin homology domain; GEF, guanine nucleotide exchange factors; p190RhoGAP, Rho GTPase-activating proteins; RacGAP, Rac GTPase-activating protein [96,99].

**Regulation of actin–myosin contractility by ANLN:** Assembly and contraction of actomyosin filaments promotes cleavage furrow formation and ingression during cytokinesis. The initiation of this furrow is caused by the activation of RhoA by Pebble (Pbl) in *Drosophila* and RhoGEF, called ECT2 in mammals. Upon the onset of anaphase, the RhoGEF ECT2 is dephosphorylated, which allows it to bind to the centralspindlin. RhoGEF ECT2–centralspindlin binds to ANLN to form a complex. This complex results in the activation of RhoA, which accumulates at the furrow. Active Rho activates several effectors. Active Rho-bound formin induces actin polymerization (F-actin) and the formation of the contractile ring. Active RhoA binds to RBD (RhoA-binding domain) of inactive ROCK and activates ROCK. Active ROCK phosphorylates the regulatory myosin light chain of myosin and the myosin-binding site of myosin phosphatase to activate myosin for furrow ingression. In this manner, these events promote the sliding of myosin heads along the actin filaments, and therefore, the formation and ingression of the cleavage furrow. When contractility force reaches a threshold, p190 RhoGAP-A effectively binds to ANLN at the cytokinesis furrow and turns RhoA-GTP into RhoA-GDP. The reduction of RhoA-GTP leads to a decrease in myosin II activation and F-actin. Finally, the release of p190 RhoGAP-A from ANLN completes one cycle. This mechanism maintains the appropriate contractility force on the actin–myosin ring for the completion of cytokinesis (Figure 3).

**Coupling of F-actin to microtubule by ANLN via interaction with RacGAP**: In order to activate Pbl/ECT2, which is involved in the activation of RhoA, this molecule requires interaction with RacGAP50C in *Drosophila* and MgcRacGAP in mammals [96]. This RacGAP molecule is one of two constituent molecules of an evolutionarily conserved complex called centralspindlin [97]. Another constituent molecule of centralspindlin is ZEN-4 in *C. elegans*, Pavarotti (Pav–KLP) in *Drosophila* and MKLP1 in mammals called a plus-end-directed kinesin-like motor protein [96].

Due to the activity of this motor component, centralspindlin in anaphase rapidly accumulates in the two types of microtubule such as the central spindle and plus-ends of the equatorial microtubule [96]. As such, the cleavage furrow ingression process seems to require signals from the spindle microtubule in the form of RacGAP signaling molecules. Consistent with this, RacGAP50C and MgcRacGAP are required for furrowing in *Drosophila* and mammals, respectively [98]. ANLN directly binds RacGAP50C to make a connection between the actomyosin filaments responsible for furrow ingression and a spindle microtubule [31].

#### 2.4.1. ANLN in Nucleus

ANLN is a contractile ring protein that cycles from the nucleus to the cell cortex via importin [12,100]. Although ANLN resides in the nucleus for a relatively long time during a cell cycle, as of now, the roles of ANLN in the nucleus have to date not been determined. Evidence has shown an association between poor tumour prognosis and highly expressed ANLN in the nucleus [8,29,101]. In addition, the downregulation of phosphoinositide 3-kinase/AKT activity in non-small cell lung cancer cells leads to the instability of ANLN and induces a reduction in the ANLN level in the nucleus [29]. This suggests that ANLN can have a role in the nucleus related to the phosphoinositide 3-kinase/AKT pathway.

Among the actin regulatory proteins, cofilin-1 and mDia2 are known to be involved in the regulation of actin in the nucleus [102]. Cofilin-1 is involved in the disassembly of nuclear F-actin in early G1, and in the case of mDia2, it seems to be involved in nuclear F-actin assembly during cell spreading, fibronectin [103] and serum stimulation [104], CENP-A loading, and nucleasome maintenance.

Especially, mDia2 bridges upstream small GTPase signaling with a downstream nuclear environment during stable CENP-A loading at the centromeres in early G1 [105]. At this time, ECT2 (GEF) and MGCRACGAP (GAP) were suggested as upstream small GTPases. These small GTPases are molecules that interact with ANLN and regulate the formation of F-actin in cytokinesis. Therefore, it seems that ANLN interacts with ECT2/MGCRACGAP/mDia2, etc., and can promote the formation of nuclear F-actin in the nucleus where these proteins are involved in the early G1 stage, and thus, affect many of the nuclear activities involved in F-actin. However, it seems that this involvement of ANLN has not been studied.

**Figure 4:**
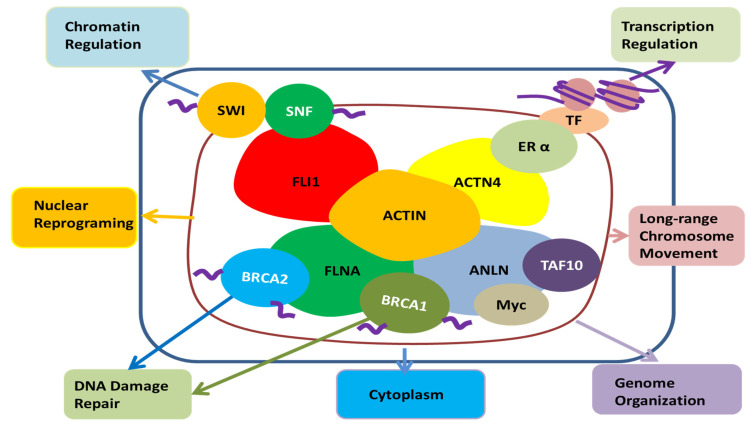
The potential role of ANLN in the nucleus; Actin present in the nucleus is combined with FLi1 homolog, α-actinin 4, filamin A, SWI/SNF, estrogen receptor α, BRCA 1, 2, etc., and it seems to be involved in chromatin remodeling, transcription regulation, nuclear reprogramming, long-range chromosome movement and DNA damage repair. ANLN has the potential to be involved in their action through binding with actin in the nucleus. Moreover, it can be involved in chromatin reorganization, transcription, and DNA damage repair through direct binding with TAF10, Myc, and BRCA1. In addition, it has been reported that ANLN is present in the nucleus to regulate the cell’s movement and infiltration by regulating the signal of the cytoplasm [114]. FLI1, friend leukemia integration 1 transcription factor; SWI/SNF, Switch/Sucrose non-fermentable; FLNA, Filamin A; BRCA1, breast cancer type 1; BRCA2, breast cancer type 2, ANLN, anillin actin-binding protein; Myc, a family of regulator genes and proto-oncogenes that code for transcription factors; TAF10, TATA-Box-binding protein associated factor 10; ACTN4, α-actinin 4; ERα, Estrogen receptor alpha; TF, transcription factor; Actin, a family of globular multi-functional proteins that form microfilaments.

Among other actin-binding proteins present in the nucleus, FLi1 homolog, α-actinin 4, and filamin A are associated with SWI/SNF, estrogen receptor α, and BRCA 1, 2, respectively, and are involved in chromatin remodeling, transcription regulation, and DNA damage repair, respectively (Figure 4) [83]. ANLN is also known to bind transcription factors such as TAF10, Myc, and BRCA1, so ANLN is likely to be involved in chromatin organization, transcription, and DNA damage repair in the nucleus, respectively [70,92]. Recent reports found that the regulation of actin polymerization in the nucleus is required for transcription activation, cell cycle progression and DNA repair [106,107]. The role of ANLN, an actin-binding protein, has yet to be determined, but it is likely to play a role in the regulation of actin monomer or polymerization processes in the nuclei.

#### 2.4.2. ANLN in Cytosol

Recently, roles other than the cell division of ANLN have been reported. That is, ANLN regulates adhesion and intercellular junction. For example, mutations in the ANLN gene cause kidney disease and focal segmental glomerulosclerosis, which indicate a defect in podosomal matrix adhesions [10]. Moreover, ANLN is a modulator of cellular cell adhesion mediated by E-cadherin in Drosophila [108]. ANLN knockdown results in abnormal adherens junctions and tight junctions in Xenopus embryos [109].

ANLN is highly expressed at the Z discs of myocardial cells, in which myosin and actin could be found as they anchored tenaciously [3]. In addition, ANLN is associated with preserving the integrity of the podocyte actin cytoskeleton [10]. Besides, ANLN has been recruited to the leading edge of migrating neuroblasts through the activation of MIG-2, a member of the Rho family of small GTPases [110]. Here, it binds active MIG-2 and stabilizes F-actin. The stabilization of F-actin requires ANLN’s ability to prohibit both actin monomer dissociation and the F-actin severing activity of cofilin. Thus, ANLN may be important for proper neuroblast migration and neuritogenesis [110].

In fact, in some cancers, ANLN is also found in the cytoplasm, suggesting the existence of nonproliferation-associated activities of ANLN [3]. Indeed, the overexpression of ANLN in the colon cancer cell lines SW480 and HT29 actually increased cell migration and invasion, which seems to be partly related to a decrease in the expression of E-cadherin [111]. In other words, it can be said that it is related to the role of ANLN in the cytoplasm. In addition, it appears that ANLN is redistributed to the leading edge of neuroblasts of *C. elegans* neurons, not cancer cells, thereby suppressing the F-actin cleavage of cofilin, stabilizing the F-actin network, and regulating cell migration and neurite growth [112].

It has also been reported to regulate breast cancer cell migration and invasion [113]. However, in this case, it seems that ANLN is present in the nucleus, which controls the movement and invasion of breast cancer cells through JNK signaling. A similar example appears to modulate the integrity of the adherens junction and the tight junction while present in the nucleus during interphase in human epithelial cancer cells DU145, SK-CO15, and A549 [114].

## 3. Role of ANLN in Tumour Cells

**Figure 5:**
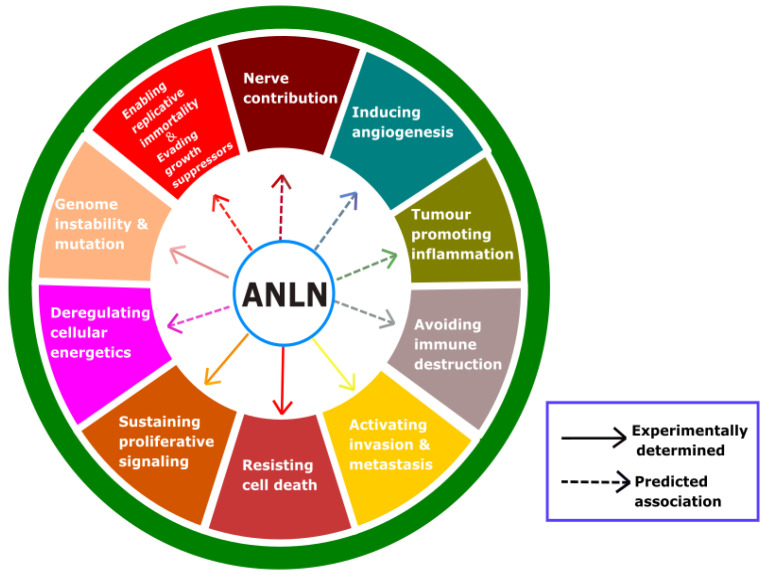
Effects of ANLN on cancer progression; this illustration encompasses eleven stimulating effects of ANLN on the hallmarks of cancer, including experimentally determined effects and predictable effects based on the association of ANLN with events related to each predicted hallmark. Separate hallmarks of cancer are depicted with differently colored fields.

To date, ten acknowledged features of cancer have been characterized, and these are referred to as the hallmarks of cancer. Based on these hallmarks, we hope that a potential target for anticancer treatment could be determined. A high upregulation of ANLN was found in several cancers (Table 2), and this protein plays an important role in cytokinesis and is a scaffold to a variety of proteins. These could suggest that ANLN should be considered as a specific target for anticancer agents. Hence, we highlight the hallmarks of cancer that are impacted by ANLN (Figure 5) and discuss them as follows.

### 3.1. What Is the Role of ANLN on Proliferation and Cell Death of Cancer Cells?

There is evidence showing that ANLN plays a critical role in driving cell proliferation [29], and the absence of ANLN could hinder cancer cells from division [115]. Besides, ANLN could be confirmed to be a Wnt/β-catenin responsive gene in gastric cancer, and it can regulate the proliferation of gastric cancer cells [122]. The analysis with flow cytometry indicated that ANLN knockdown in MDA-MB-231 cells inhibited the cell cycle progression, with an increasing amount of cells stuck at the G2/M phase because of phosphorylation of Cdc2 and an inhibition of Cyclin D1 [106]. Moreover, cyclin D1 was reported to have a relationship with apoptosis in response to γ irradiation in breast cancer cell lines [123]. ANLN was recorded to be involved in apoptosis in hepatocellular carcinoma (HCC).

ANLN deletion led to an increase in polyploidy cells along with the activation of apoptosis and DNA damage [116]. In pancreatic cancer, ANLN deficiency led to the expression of miR218-5p while mir-218 caused the apoptosis of pancreatic cancer cells [119]. This indicates that the reduction of ANLN is indirectly associated with the induction of apoptosis. ANLN was known to be involved in PI3K/PTEN signaling [59]. The knockdown of ANLN suppressed cell proliferation and induced apoptosis in nasopharyngeal carcinoma (NPC) cells [117].

In addition, the role of ANLN in bladder urothelial carcinoma growth was tested on J82 and 5637 cells by making a comparison between ANLN knockdown and control groups. When it comes to apoptosis, no significant difference was recorded [118]. ANLN deficiency critically decreased cell proliferation and colony formation in nude mice [5].

### 3.2. What Is the Role of ANLN on Invasion and Metastasis of Cancer Cells?

ANLN deficiency critically decreased cell migration and invasion [5]. ANLN downregulation substantially suppressed the migration of breast cancer cells [106]. Moreover, the significant role of ANLN in cell mobility has been confirmed by Matrigel of NIH3T3 and COS-7 cells transfected with ANLN expression vectors and wound-healing assays using NIH3T3 cells transfected with ANLN plasmids or mock plasmid [29].

ANLN expression was significantly upregulated in pancreatic cancer tissues and cell lines, and a high upregulation was implicated with lymph node metastasis, distant metastasis, and poor prognosis in pancreatic cancer. ANLN silencing could lead to migration suppression in NPC cells [117]. In addition, ANLN downregulation induced the inhibition of some cell–cell adhesion related genes, such as the gene encoding LIM and SH3 protein 1 (LASP1) [5]. These pieces of evidence underpin the association between ANLN and cell migration.

In addition, the incidence of metastasis in patients with a high ANLN expression was substantially higher than that in patients with low expression. Nevertheless, ANLN downregulation led to an E-cadherin and vimentin expression increase and N-cadherin decrease in A549 and PC9 cells, which results in a decrease in the migration and invasion ability of A549 and PC9 cells. These findings suggest that ANLN upregulation in lung adenocarcinoma is implicated with the metastasis of cancer cells. ANLN may be involved in the metastasis of lung adenocarcinoma by promoting the epithelial mesenchymal transformation of tumour cells [120].

ANLN knockdown in J82 and 5637 cells, by small interfering RNA, substantially prohibited cell migration and invasion ability. Besides, a microarray analysis revealed that ANLN plays a key role in cell migration [118].

### 3.3. What Is the Role of ANLN in Replicative Immortality and DNA Repair?

There is not much evidence regarding the impact of ANLN on miR-497. However, ANLN was considered to be an exclusive target of miR-497 [117]. The transfection of a miR-497 mimic into NPC cells inhibited cell growth and caused apoptosis. Besides, ANLN was highly regulated in NPC cells that were subjected to the substantial downregulation of ANLN by exogenous miR-497 [117,124]. The evidence indicates that miR-497 could be a potent growth suppressor by targeting ANLN. Moreover, p53 is a growth suppressor in cancer cells and ANLN is known as one of the genes regulated by the p53–DREAM pathway [125]. The evidence indicates that miR-497 could be a potent growth suppressor by targeting ANLN. Replicative immortality is one of the important features of tumour cells. Cells that show the upregulation of telomerase and inactive suppressors are resistant to cell growth arrest [126]. To date, there are no findings on the direct effect of ANLN on telomere or telomerase. However, ANLN and telomerase are both genes related to cell cycle arrest through p53 [126]. More compellingly, Myc is an interactor with ANLN that plays an important role in activating the expression of telomerase [127]. This could imply that ANLN could manipulate the expression of telomerase via the expression of Myc.

ANLN was considered as one of 17 markers for global genomic instability in breast cancer [128]. DNA damage is a cause of genome instability [129]. The deficiency of ANLN in U2OS cells progressively induced an increase in the number and intensity of 53BP1 foci in G1 nuclei, a phenomenon that could act as a marker for an increased number of DNA damage events [121]. Which could suggest that ANLN could be associated with the stability of the genome. ANLN interacts with BRCA1 [92], so aberrant ANLN expression might affect genome instability and mutation.

### 3.4. Possible Role of ANLN on Other Hallmarks of Cancer

**Cancer metabolism**: Cancer metabolism is a novel mechanism considered for cancer treatment [130,131]. ANLN knockdown was documented to induce polyploid hepatocytes [132], which induce an upregulation of the genes controlling lipid metabolism and a downregulation of the genes controlling mitochondrial oxidation [133]. ANLN could be a component of the PI3K/Akt pathway [29] that plays a significant role in the metabolism of insulin-mediated glucose [134] or in metabolic reprogramming in terms of glucose, glutamine, nucleotides and lipids in cancer [135]. These findings suggest that ANLN could be involved in mediating cancer metabolism.

**Importance of tumour microenvironments**: Cancer treatments addressing the tumour microenvironment have been noted as essential, since the microenvironment itself contributes to cancer heterogeneity [136]. Tumour microenvironments could encourage or hinder cancer [137]. Hence, the re-education of the microenvironment from promoting to inhibiting is an action that is needed [138,139]. Recently, there has been research related to reforming the microenvironment, such as with immune checkpoint inhibitors [139]. Three characteristics of the hallmarks of cancer—inducing angiogenesis, promoting inflammation, and avoiding immune destruction—contribute to the manipulation of the tumour microenvironment. There is not much evidence of the direct effects of ANLN on re-educating the tumour microenvironments, but ANLN is a multiple-domain scaffold for many proteins, which suggests that ANLN could indirectly regulate the microenvironment through interactors.

**Angiogenesis:** Angiogenesis is one of the requirements for tumour growth and metastasis [140,141]. To date, there is not much evidence of the impact that ANLN expression has on angiogenesis. However, VEGFR-2 (also known as kinase insert domain receptor or KDR) is known to be a genetic interactor of ANLN [59] that promotes angiogenesis [142,143]. Moreover, there is an association between PI3K/AKT and the stability of ANLN in the nucleus [29] and the activation of PI3K/AKT in the tumour could increase the VEGF level [144]. Taken together, these findings may suggest that there is an association between ANLN and angiogenesis.

**Inflammation:** Inflammation contributes to cancer progression and development [145,146]. In the early stage of neoplasm, cancer cells and the surrounding stromal and inflammatory cells could act as robust tumour promoters by jointly generating a compatible environment, called the inflammatory tumour microenvironment (TME) for tumour thriving, angiogenesis and genomic instability [146]. In the late stage of the tumourigenic process, neoplastic cells have an impact on the inflammatory mechanisms, such as chemokine functions, to bolster tumour spread and metastasis [146]. As of now, there is slight evidence of the association between ANLN and inflammation. In a study on Dalmatian dogs, the loss of ANLN caused a familial fatal acute respiratory distress syndrome [147], which is a disease where inflammatory mediators such as IL-8, IL-6 are suggested as the potential culprits [147,148,149]. Besides, ANLN is likely to be correlated with tumour necrosis factor (TNF) [149], and TNF-α is known as a biomarker of inflammation [150,151]. Moreover, the incubation of primary human endothelial cells with IFN-γ increased the transcription of ANLN [152]. Although this relation is unclear, it suggests that ANLN and inflammation perhaps have an impact on each other. This association should be studied further.

**Immune evasion**: The immune system could be either a friend or an enemy of cancer. Immune evasion is a term used when tumour cells circumvent a tumour-suppressive immune system [153]. Cancer cells have different strategies to evade immunity, and several cancer cells shun surveillance by downregulating the expression of antigen-presenting proteins at the plasma membrane, allowing them not to be arrested by cytotoxic T lymphocytes [154,155]. It is common for tumours to produce cytokines to manipulate immunity by prohibiting effector T cell responses and stimulating suppressive regulatory T cells [154,156]. There is not much direct evidence of the relation between ANLN expression and the immune evasion of tumours. As mentioned above, ANLN overexpression increases the efficacy of non-muscle myosin II by coordinating between myosin and bundled F-actin [1]. Interestingly, high myosin II activity in tumour cells could reprogram the innate immune microenvironment to fortify tumour growth [157]. In addition, there was an immune complex containing both ANLN and Rho, detected by a Western blot analysis by utilizing antibodies raised against either of the proteins [54]. Another study also suggests a critical role in the immune response through a synergy between ANLN and KDR, which have prognostic value in breast cancer survival [59].

**Nerve connection**: The success of immune checkpoint drugs has confirmed the importance of the tumour microenvironment in the treatment area of cancer, and the importance of nerves associated with cancer has also recently been highlighted [158,159]. Many reports have shown that nerve elements are involved in tumour progression. For example, almost all types of peripheral cancer have been observed to interact with neural structures, at least in advanced stages, especially in bladder cancer, prostate cancer, pancreatic cancer, colon cancer, lung cancer, head neck cancer, bile duct cancer, and glioma [158,160,161]. In addition, with a decrease in the chronic-stress-induced sympathetic nerve activation and tumour innervation density, recurrence-free survival is shown to be higher [162,163].

ANLN facilitates septin assembly to prevent pathological outfoldings of central nervous system myelin [164]. ANLN is required by Schwann cells (SCs) for axonal fragmentation [165]. Septin/ANLN filaments scaffold central nervous system myelin to accelerate nerve conduction [166]. SEPT7 interacts with KIF20A and regulates the proliferative state of neural progenitor cells during cortical development [167]. RhoG signals through the multi-domain protein ANLN to stabilize F-actin in these structures [110,112]. Therefore, considering the role of the ANLN in the nerve, it is expected that ANLN will also play a role in contributing to the nerve elements in tumour progression.

## 4. Biomarker and Potential Therapeutic Options

### 4.1. ANLN as a Biomarker

Since it is essential for cell division, ANLN is critical in the development and homeostasis of mammalian cells. ANLN was upregulated in many distinct tissues (Table 3). ANLN expression levels correlate with the metastatic potential of human tumours from many different tissue origins as follows.

The inhibition of ANLN expression suppressed the growth of lung cancer cells in culture. Considering that human ANLN is normally degraded after a mitotic exit and sequestered in the nucleus during interphase, its overexpression may overwhelm these normal regulatory mechanisms, freeing ANLN to have an impact on the actin–myosin cytoskeleton during the events besides cytokinesis, including, for example, cell motility, and thus it directly contributes to cancer progression.

To date, there is much evidence underpinning the association of ANLN to cancer development. ANLN expression was documented to be upregulated from ~2- to 6-fold in cancer tumours compared to normal ones, except brain tumours. In one study, a highly-expressed ANLN protein was proven to be implicated in the metastatic risk of tumours [3]. In another research about pancreatic cancers, the ANLN level of pancreatic tumours was recorded to increase 20-fold in comparison with normal tissues [4]. ANLN induced EZH2 upregulation and is involved in pancreatic cancer progression by mediating the miR-218-5p/LASP1 signaling axis, EZH2 upregulation or miR-218-5p downregulation, and LASP1 partially contributes to reversing the anti-tumour effect of ANLN deficiency on pancreatic cancer cell development [5]. The regulation of actin-binding protein ANLN through anti-tumour miR-217 suppressed cancer cell aggressiveness in pancreatic ductal adenocarcinoma [6]. In addition, ANLN overexpression could be a cause that leads to an increase in cytosolic levels in interphase cells. This may contribute to cell shape changes and to cell motility, as well as metastasis. There are several papers supporting this hypothesis. Upregulated ANLN was also recorded to reside in the ectopic foci that involves RhoA and septins [16,22].

In other research, there is evidence showing that ANLN is also related to metastasis in lung adenocarcinoma (A549, PC9). The inhibition of ANLN in A549 and PC9 by ANLN siRNA could have an impact on EMT, which contributes to cell migration and invasion [9,120]. Besides, ANLN plays a role in human lung carcinogenesis via the activation of RhoA and by involvement in the phosphoinositide 3-Kinase/AKT Pathway [29]. The Cancer Genome Atlas data analysis revealed 27 mutations in 446 patients with lung adenocarcinoma, with five mutations affecting the conserved amino acids of ANLN [9].

Knockdown of ANLN by lentivirus inhibited breast cancer cell growth, and ANLN in primary breast cancer was documented as a potential biomarker of Ki-67, which substantially contributes to cell progression [8,106].

Additionally, ANLN is involved in regulating the cell growth, migration, and metastasis of breast cancer [106]. Consistently with the above study, ANLN and KRD were also investigated as jointly prognostic of breast cancer [71].

Based on the analysis of colorectal cancer tissues, ANLN is supposed to be associated with colorectal cancer development and with a poor prognosis [7]. ANLN upregulation was also observed in hormone-refractory prostate cancers (HRPCs) [26]. An integrated bioinformatics analysis showed that ANLN could play a role as a key candidate in cervical cancer [34]. Transcriptome sequencing identified ANLN as a potential prognostic biomarker in bladder urothelial carcinoma [118]. Based on in silico and in vitro data, ANLN and TLE2 are shown to be potential biomarkers for muscle invasive bladder cancer [168]. ANLN was found as a target of head and neck squamous cell carcinomas via genome-wide gene expression profiling [169].

As described above, ANLN is localized in both the nuclei and cytosol. Although its function was unclear, nucleus ANLN was supposed to play a role in a poor cancer prognosis [1,29]. Moreover, the roles of ANLN in cancer development in both cytokinesis and outside should be further characterized.

**Table 3:** ANLN overexpression in the different tissue types [170].

Tissue	Samples Overexpressed/Total Sample Tested	Percentages of Samples Overexpressed (%)
Adrenal gland	4/79	5.06
Breast	125/1104	11.32
Central nervous system	30/697	4.3
Cervix	19/307	6.19
Endometrium	45/602	7.48
Hematopoietic and lymphoid	10/211	4.52
Kidney	33/600	5.5
Large intestine	29/610	4.75
Liver	25/373	6.7
Lung	148/1019	14.52
Oesophagus	18/125	14.4
Ovary	12/266	4.51
Pancreas	15/179	8.38
Prostate	46/498	9.24
Skin	43/473	9.09
Soft tissue	23/263	8.75
Stomach	32/285	11.23
Thyroid	28/513	5.46
Upper respiratory tract	54/522	10.34
Urinary tract	34/408	8.33

### 4.2. Potential Therapeutic Options

As of now, there are not many reports on ANLN inhibitors. However, some studies have shown that both total ANLN and p-ANLN expression decreased by LY294002 (Sigma-Aldrich; 20 μmol/L for 16 h) [29], a specific inhibitor of the catalytic subunit of PI3K, which is directed at the ATP-binding site of the kinase [171]. Calcineurin (Cn) inhibitors such as VIVIT-a peptide inhibitor that could disrupt the interaction between Cn and nuclear factor of activated T-cells [172], reduced the expression of ANLN in podocyte cells [173].

**Figure 6:**
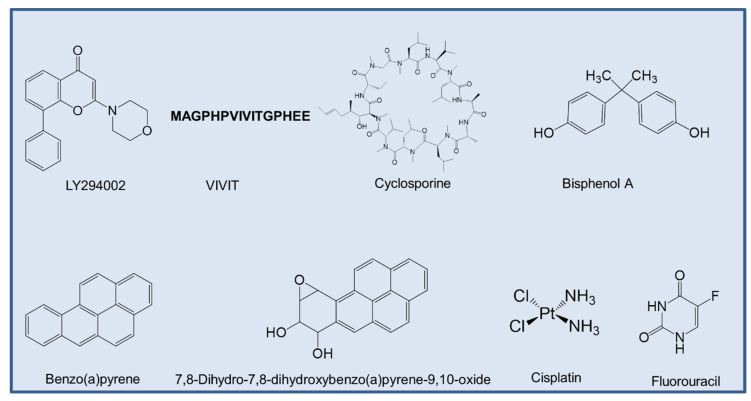
The potential inhibitors of ANLN.

Based on the data derived from the Comparative Toxicogenomics Database (CTD) [174], cyclosporine, bisphenol A, benzo(a)pyrene, 7,8-dihydro-7,8-dihydroxybenzo(a)pyrene 9,10-oxide, cisplatin, fluorouracil were curated, all of which are chemicals that could inhibit the expression of ANLN mRNA (Figure 6). Among the aforementioned compounds, some of them have experimental proof to show their effects in cancer treatment.

Cyclosporine is a natural product that is used as an immunosuppressive drug [175]. Cyclosporine A has been recorded to suppress breast cancer growth by downregulating the expression of pyruvate kinase subtype M2 [176].

Cisplatin is an approved chemotherapy to treat a variety of cancers, including ovarian cancer, bladder cancer, and lung cancer [177]. The main mechanism of action in cancer treatment is to induce apoptosis in cancer cells by a crosslink with the purine bases on the DNA, and then interfering DNA repair mechanisms, subsequently leading to DNA damage [177].

Fluorouracil is well known for its use in cancer treatment, especially for colorectal cancer [178]. The mechanism of the cytotoxicity of 5-FU has been depicted to cause the misincorporation of fluoronucleotides in RNA and DNA, and the suppression of the nucleotide synthetic enzyme thymidylate synthase [178]. However, the direct effect of these compounds on ANLN has not been determined and should be further studied to determine which one is specific to suppress ANLN expression.

Considering the role of ANLN in normal cells, it is worth considering the potential toxicity of inhibiting ANLN. ANLN mRNA is lowly expressed in most immune cell types in blood, but T-reg highly expressed ANLN in its RNA level [179]. These findings suggest that ANLN is maybe essential for the proliferation of T-reg cells. Moreover, T-reg is known to be involved in tumour development and progression by suppressing anti-tumour immunity [180]. Therefore, the effect of ANLN on immune cells may not be as great as expected. Of course, it plays an important role at the moment of proliferation of blood cells in the bone marrow. In the case of normal cells, the number of times proliferation occurs is more limited than that of cancer cells, so the toxic effect of suppressing ANLN is thought to be limited. In support of this idea, there has been a recent report that ANLN knockdown lowers the incidence of liver cancer but does not affect liver regeneration [115]. Besides, an appropriate drug delivery system might be an answer to overcome the possible limitation of ANLN inhibitors which may achieve high local concentrations of the drug in the target area with low side effects in normal tissues.

## 5. Perspectives

We have introduced a concise summary of ANLN as a biomarker and as a novel target of cancer development. Although many studies have shown the impact of ANLN on several hallmarks of cancer, some hallmarks have not yet been studied much. ANLN is overexpressed in many types of cancers, and there are several studies showing that the upregulation of ANLN is associated with cancer development.

Evidence indicates that ANLN still has roles outside cytokinesis, and these roles are maybe related to cancer development. However, the association between ANLN and these partners in cancer development has not been fully uncovered to date, and its role in the nucleus remains unknown. In particular, the discovery of ANLN as a binding partner of DNA repair molecule BRCA1 and KIAA1429 and CDC5L, which are involved in mRNA splicing, suggests that ANLN may play a role in DNA repair and mRNA splicing. It is expected that the role of ANLN in this phenomenon will be studied in the future. We feel the need for an understanding of the roles of ANLN, other than its acknowledged roles in cytokinesis. Thus, these novel roles need to be studied further in its aspects of normal physiology and pathophysiology.

The value of ANLN as a biomarker in cancer seems to be high, and the development of ANLN inhibitors by considering ANLN as a therapeutic option in cancer seems to be worth further consideration. To the best of our knowledge, there is no specific ANLN inhibitor that has been found to date. It is imperative to discover compounds that could specifically inhibit ANLN, because these putative ANLN inhibitors could help deeply understand the role of ANLN in events outside cytokinesis, and its interactions with oncoproteins in cancer development. Another way to inhibit ANLN may be to further elucidate the mechanism of action of regulatory molecules, such as kinase, for the regulation of ANLN to suppress that regulatory mechanism. For example, ANLN contains phosphorylation sites, and S635 was confirmed to have an important role in cytokinesis completion. However, which kinases are responsible for phosphorylating ANLN remains unknown. These kinases may be a potential target for tumour treatment because cytokinesis is necessary for tumour growth. Other novel approaches such as the removal of ANLN by PROTAC approach and protein–protein inhibitor are also possible.

Studies of the ANLN-specific inhibitor as well as of the role of ANLN in cancer development, especially its role outside the cytokinesis event, are of paramount importance and could bring many benefits to cancer treatment.

## Figures and Tables

**Table 1:** The roles and characteristics of ANLN and its partners in cytokinesis.

Partners of ANLN	System	Binding Regions of ANLN	Binding Regions of Partners	Ref	Role of ANLN to Partners	Characteristics in the Association between ANLN and the Partners
Actin	*Drosophila*	Amino acids 258–340	Full length	[12]	- Amino acid 246–371 Drosophila ANLN could bundle F-actin [1,12]	- Specifically bind to F-actin, but not enrich around G-actin [18]. - ANLN specifically binds to cell division-related contractile F-actin, not all F-actin in the cells [1]. - F-actin and ANLN F-actin and ANLN are independently attracted to the contractile ring in human and *Drosophila* cells [13,19,20,21]. - F-actin has a role in supporting ANLN to locate in the cell equator with accurate timing and spatial positioning [13,21,22].
	Human	Amino acids 231–454	Full length	[22]		
	*X. Leavis*	Amino acids 255–418	Full length	[23]		
Septins (a group of GTP-binding proteins)	Human	Amino acids 929–1125	Full length	[22]	- In *C. elegans*, septins are recruited by ANLN to the contractile ring [16,24] - Promote furrow ingression [1]	- *C. elegans* ANLN recruitment in the contractile ring was independent of septin enrichment [25].
	Human	Amino acid 748–1116	Full length	[23]		
	*Drosophila*	Amino acids 815–1201	Full length	[26]		
Myosin	*X. Leavis*	Amino acids 142–254	Full length	[27]	- The main function of ANLN to myosin is to organize them at actin–myosin ring [28]	- ANLN (ANI-1 in *C. elegans*) and myosin are attracted to the contractile ring independently [13,25,27]. - *C. elegans* ANLN (ANI-2) is required for the integrity of the myosin [25].
RhoA	Human	Amino acids 608–943	Full length	[16]	- Stabilize RhoA and link RhoA to the contractile ring [16]	- The upregulation of ANLN could induce a significant increase in the rate of active RhoA [29].
RacGap	*Drosophila*	Amino acids 517–1212	Amino acids 83–309	[30]	- Crosslink the central spindle to the contractile ring [31]	- ANLN and Racgap co-expressed at the plus ends of microtubules during cytokinesis [30,31].
	*Drosophila*	Amino acids 929–129	Amino acids 136–371	[31]		
Ect2	Human	Amino acids 926–980	Full length	[1]	- Stabilize RhoA activation and cortical localization of central spindle proteins [32]	- ANLN’s interaction with the Ect2 complex requires Ect2’s association with phospholipids [32]
	Human	Amino acids 421–621	Amino acids 608–940	[32]		
CD2A	Human	Amino acids 1–155	Amino acids 1–175	[33]	- Stabilize intercellular bridge [33,34]	- PX(P/A)XXR motif required for the interaction of ANLN with CD2AP [33]. - ANLN and like CD2AP are phosphorylated in mitosis, and the dephosphorylation of both happens in a similar time course [33]. - Cindr colocalizes with ANLN at stable somatic intercellular bridges [34].
	*Drosophila*	Amino acids 1–328	Full length	[34]		
	*Drosophila*	Amino acids 930–1239	Full length	[34]		
Microtubules	*Drosophila*	Full length	Full length	[35]	- Crosslink cleavage furrow with microtubules to stabilize the furrow [35]	- ANLN-rich structures colocalize with the plus ends of microtubules [1].

**Table 2:** The role of ANLN on the hallmarks in different cancer cell lines.

Hallmarks of Cancer	Cancer Type	Cancer Cell Line	Effect	References
Proliferation	Liver cancer	H2.35, QGY-7703, BEL-7404, Hep3B, MHCC-97L, Huh7, HepG2, PLC/PRC/5, BEL-7405, HepG2.215, SMMC-7721 and Sk-Hep-1	Upregulate	[115,116]
	Lung cancer	A549, LC319, PC-3, PC-9, PC-14, A427, NCI-H1373	Upregulate	[29]
	Nasopharyngeal carcinoma	HK1, CNE1, HONE1	Upregulate	[117]
	Bladder urothelial carcinoma	J82, 5637	Upregulate	[118]
	Pancreatic cancer	BxPC-3, SW1990	Upregulate	[5]
	Breast cancer	MDA-MB-231	Upregulate	[106]
Apoptosis	Breast cancer	MDA-MB-231	Downregulate	[106]
	Liver cancer	H2.35, QGY-7703, BEL-7404, Hep3B, MHC C-97L, Huh7, HepG2, PLC/PRC/5, BEL-7405, HepaG2.215, SMMC-7721 and Sk-Hep-1	Downregulate	[116]
	Pancreatic cancer	AsPC-1, BxPC-3, and PANC-1	Downregulate	[119]
	Nasopharyngeal carcinoma	HK1, CNE1, HONE1	Downregulate	[117]
	Bladder urothelial carcinoma	J82, 5637	No effect	[118]
Invasion and metastasis	Pancreatic cancer	BxPC-3, SW1990	Upregulate	[5]
	Breast cancer	MDA-MB-231	Upregulate	[106]
	Lung cancer	NIH3T3, COS-7, A549, PC9	Upregulate	[29,120]
	Bladder urothelial carcinoma	J82, 5637 cells	Upregulate	[118]
	Nasopharyngeal carcinoma	HK1, CNE1, HONE1	Upregulate	[117]
Growth suppressor	Not experimentally determined			
Cell immortality	Not experimentally determined			
Genome instability and mutation	Bone cancer	U2OS	Downregulate	[121]
Cancer metabolism	Not experimentally determined			
Angiogenesis	Not experimentally determined			
Inflammation	Not experimentally determined			
Immune evasion	Not experimentally determined			
Nerve connection	Not experimentally determined			
